# Left ventricular myxoma: case report

**DOI:** 10.11604/pamj.2020.36.358.24793

**Published:** 2020-08-27

**Authors:** Mohamed Rida Ajaja, Amine Cheikh, Noëllie Akpabie, Wafa Elmire, Amale Tazi Mezalek, Amine El Hassani, Mahdi Ait Houssa

**Affiliations:** 1 Department of Cardiac Surgery, Cheikh Zaid Hospital, Abulcasis University, Rabat, Morocco,; 2 Department of Pharmacy, Cheikh Zaid Hospital, Abulcasis University, Rabat, Morocco,; 3 Department of Cardiology, Cheikh Zaid Hospital, Abulcasis University, Rabat, Morocco,; 4 Department of Pediatrics, Cheikh Zaid Hospital, Abulcasis University, Rabat, Morocco

**Keywords:** Left ventricular myxoma, echocardiography, cardiopulmonary bypass, resection

## Abstract

Left ventricular (LV) myxomas are rare. We present a case of a LV myxoma arising from the interventricular septum in a 70-year-old asymptomatique man. General examination of the patient did not reveal any abnormality. Transthoracic echocardiography revealed a round pedunculated mass (size, 20mm x 13mm) at the interventricular septum with a broad pedicle. The mass was successfully removed and was pathologically confirmed to be a myxoma.

## Introduction

Cardiac myxomas are the most common benign cardiac tumors, accounting for 80% of cases in reported series [1]. Cardiac myxomas are typically atrial in origin [2]. Myxomas usually have solid or papillary pattern when located in an atypical position [3]. They are uncommon tumors with an annual incidence of 0.5 per million individuals. Furthermore, left ventricle (LV) myxomas are rare, accounting for only 1.7% of all cardiac myxomas [4]. We present a case of an LV outflow tract myxoma that was diagnosed using echocardiography, was treated surgically and confirmed pathologically.

## Patient and observation

**Figure 1:**
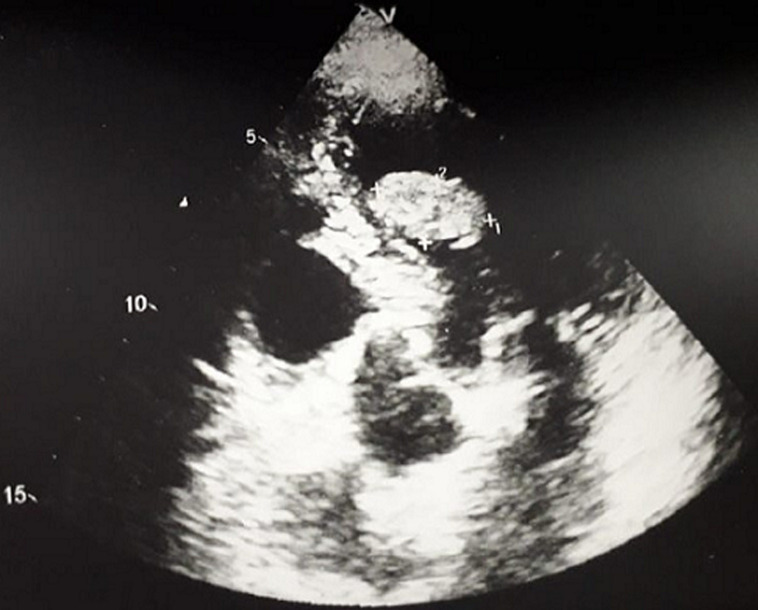
echocardiogram demonstrating a left ventricular mass in a 62-year-old asymptomatic male patient

**Figure 2:**
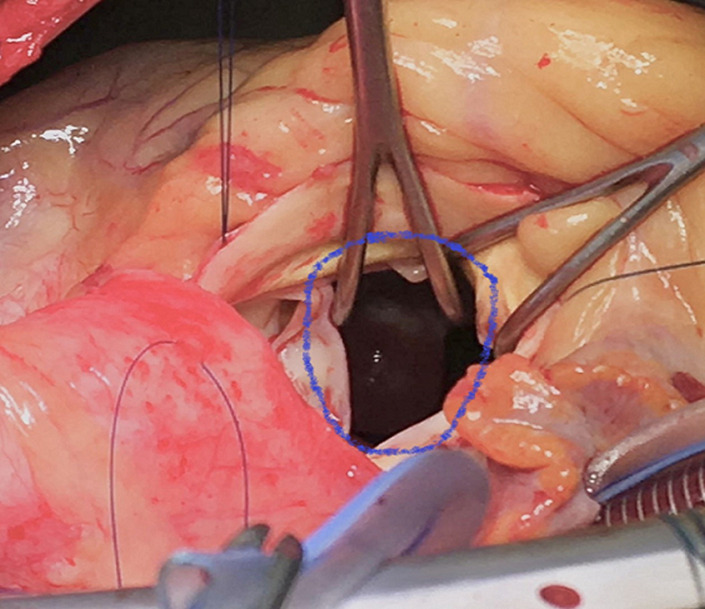
intraoperative findings: the mass pulled through the aortotomy

**Figure 3:**
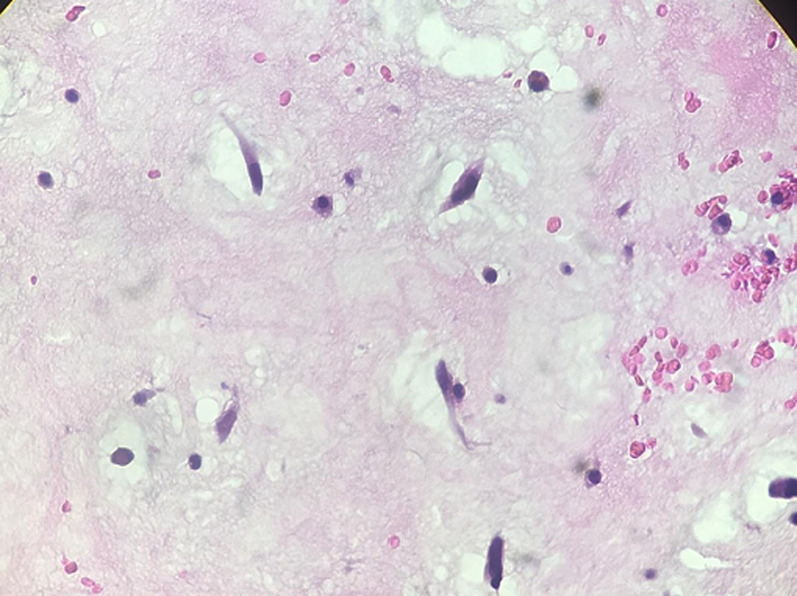
histopathologic examination confirms the diagnosis of myxoma (H&E, x40)

A 70-year-old man with dyspnea on exertion. He is followed for arterial hypertension. The patient had no history of any cardiac disorders or any family history of cardiac masses. On physical examination, her heart rate was 50bpm and her blood pressure was 140/70mmHg. Chest radiograph revealed a cardiothoracic ratio of 55% with no evidence of pulmonary edema or metastasis. An electrocardiogram revealed sinus rhythm with a heart rate of 50bpm. Laboratory tests were normal limits. Transthoracic echocardiography revealed a round, pedunculated, mobile mass (size, 20mm X 13mm) in the LV outflow tract that seemed to arise from the LV side of the interventricular septum, but was without calcification, hyperlucency, or vasculature (Figure 1). The other cardiac valves and cavities were free of lesions. The remaining echocardiographic findings were normal with an LV ejection fraction of 60%. Angiographic study revealed normal coronary arteries. The LV mass was surgically excised under cardiopulmonary bypass via a median sternotomy. The ascending aorta and both venae cavae were cannulated and standard cardiopulmonary bypass was performed. The heart was stopped by cross-clamping the ascending aorta. Myocardial protection was achieved by means of tropical cooing with ice and intermittent anterograde administration of cold-blood cardioplegic solution. After aortotomy, a jelly-like mass became visible in the LV outflow tract through the aortic cusps (Figure 2). The LV mass was completely removed with adjacent tissue of the interventricular septum. The results of the histopathologic examination of the excised specimen were consistent with the diagnosis of a myxoma (Figure 3). The postoperative course of the patient was uneventful and she was discharged without any complications.

## Discussion

Cardiac myxoma is the most common type of benign cardiac tumor, accounting for 30-50% of all primary heart tumors. A meta-analysis indicated that 80% of cardiac myxomas occur in the left atrium, whereas LV myxomas account for 1.7% [1]. In spite of the benign histological character of these tumors, they can lead to unfavorable consequences. These tumors could aggravate preexisting complications or could even lead to sudden death due to their fragmentatibility or valve obstruction [5-7]. The cardiac symptoms caused by myxoma of the LV include syncope, dyspnea, embolic events, arrhythmias, or collapse [8]. The most common primary tumors are rhabdomyoma, myxoma and papillary fibroelastoma. The shape, the extension, the site of attachment, the involvement of valve leaflets and the functional obstruction of the LV outflow tract can be promptly and easily assessed using echocardiography. Surgical treatment should be performed as soon as the diagnosis is confirmed even through there were no signs of embolism and obstruction. The selection of surgical incision for left ventricular myxoma depends on its location. In this case, the myxoma adhered to the base of the anterolateral papillary muscle and moved freely in the left ventricular outflow tract. Therefore, we made the aortic incision and could see the myxoma easily. Robert *et al*. chose a transmitral procedure and found that the mitral valve and its chordae tendineae could not be preserved at its location [5]. Arruda *et al*. removed a left ventricular myxoma with a 4cm incision in the left ventricular lateral wall [7]. However, in our opinion, it is better to avoid ventriculotomy due to its potential complications. Furthermore, if necessary, exploration via the right atrium and atrial septum may be performed, so aorto-bicaval cardiopulmonary bypass is better. Careful handling of the cardiac structures and tumor during its removal reduces the possibility of fragmentation and the occurrence of embolic phenomenon during the surgery. The resection of the base of the tumor implantation should be performed with good safety margin to avoid recurrence. Most importantly, care should be taken not to injure the valve, chordae tendineae and papillary muscle.

## Conclusion

Cardiac myxomas are rare tumors specifically myxomas of the left ventricle. Surgical treatment remains the benchmark in the treatment of myxomas. Resection of an LV myxoma depends on the location and if the tumor arises high enough from the ventricular septum, a transaortic approach is preferable.
